# Identification of chlorophyll a-b binding protein AB96 as a novel TGFβ1 neutralizing agent

**DOI:** 10.1038/s41598-021-87454-x

**Published:** 2021-04-08

**Authors:** Steven Lynham, Fabio Grundland Freile, Natasha M. Puri, Nicola O’Reilly, Graham H. Mitchell, Timothy N. C. Wells, Merlin Willcox, Richard Beatson

**Affiliations:** 1 Centre of Excellence for Mass Spectrometry, Denmark Hill, King’s College London, London, UK; 2 School of Cancer and Pharmaceutical Sciences, King’s College London, London, UK; 3 Peptide Chemistry Science Technology Platform, CRICK, London, UK; 4 School of Life Sciences, University of Essex, Colchester, UK; 5 Medicines for Malaria Venture, Geneva, Switzerland; 6 School of Primary Care, Population Sciences and Medical Education, University of Southampton, Southampton, UK

**Keywords:** Immunology, Cytokines, Immune evasion, Infectious diseases, Infectious diseases

## Abstract

The discovery of compounds and proteins from plants has greatly contributed to modern medicine. *Vernonia amygdalina* Del. (Compositae) is used by humans and primates for a variety of conditions including parasitic infection. This paper describes the serendipitous discovery that *V. amygdalina* extract was able to bind to, and functionally inhibit, active TGFβ1. The binding agent was isolated and identified as chlorophyll a-b binding protein AB96. Given that active TGFβ1 contributes to the pathology of many infectious diseases, inhibiting these processes may explain some of the benefits associated with the ingestion of this species. This is the first plant-derived cytokine-neutralizing protein to be described and paves the way for further such discoveries.

## Introduction

*Vernonia amygdalina* Del. (Compositae) (VA) is used by humans and other primates as a natural remedy for a variety of pathologies, including plasmodium and helminth infections^1–3^. It is commonly used throughout sub-Saharan Africa and is normally consumed as a decoction or tea made with boiling water, although in some cases it is consumed raw. There is a large body of ethnobotanic evidence supporting this claimed activity^2,3^. Unusually for ethnobotanic therapies, this is supported by multiple studies in preclinical models of parasite infection using standardized VA preparations for both plasmodium^4–6^ and helminth infections^7,8^. A clinical trial in malaria patients also confirmed that the aqueous extract has significant anti-parasitic activity^9^. The anti-infective activity has been confirmed in the purified sesquiterpene lactone, vernodalin^2^, however this molecule displays low selectivity and can cause host cytotoxicity^10^.

TGFβ1 is a highly conserved pleiotropic cytokine that is involved in multiple healthy and disease processes, including immune regulation, fibrosis, apoptosis, angiogenesis and development. TGFβ1 exists predominantly in its latent form at high concentrations throughout the body. The latent form consists of either TGFβ1 non-covalently bound to LAP (Latency Associated Peptide) forming the small latency complex, or the small latency complex bound to LTBP1-4 (Latent TGFβ Binding Protein 1-4), which forms the large latency complex^11^. Activation of TGFβ1 can be induced through several mechanisms including acidification and heating in vitro or integrin engagement, thrombospondin interaction, protease cleavage, mild mechanical or pH stress in vivo^11^.

From an immune perspective, its latent form is largely inert. However in its active form it can (a) induce regulatory T-cells (Tregs) which suppress the function of other immune cells to limit the immune response, (b) induce class switching of B cells from IgG to IgA producers, with IgA inducing less inflammatory responses (c) induce tolerogenic antigen presenting cells, which promote anergy/non-response to antigen and (d) inhibit the polarization of T helper (CD4) cells^12^ resulting in an impaired immune response. These processes are commonly seen in cancers, indeed TGFβ1 signaling is currently being targeted in > 50 cancer clinical trials through the use of antibodies or specific inhibitors^13^.

As discussed, VA is commonly used by humans and primates when infected by plasmodium or helminth parasites. During plasmodium infection, active TGFβ1 can be seen to peak 48 h after infection in sera, owing to cleavage of LAP by specific plasmodium-derived enzymes^14^. This activation can be seen to correlate with the induction of Tregs^15^, which facilitate plasmodium growth^16^. It is worth noting at this point that individuals of Fulani heritage in West Africa frequently carry a mutation which prevents Treg induction and have reduced susceptibility to *P. falciparum* infection^17^.

In intestinal helminth infection, TGFβ1 is induced and activated by the parasite inducing a profound increase in Tregs^18^, to such an extent that controlled infection is being trialed for a number of autoimmune diseases including Graft versus Host Disease (GvHD)^19^. Interestingly beyond immune evasion, through TGFβ1 induction and activation within the host, it has recently been shown that the helminth, *Heligmosomoides polygyrus,* produces its own functional TGFβ1 mimetic^20^, whilst in *Schistosoma mansoni*, human TGFβ1 directly regulates parasitic gene expression^21^, suggesting the critical importance of this pathway for parasitic survival.

Chlorophyll a-b binding protein (CabBP) AB96 is a component of the light harvesting complex of photosystem II, and is found in the thylakoid membrane of photosynthesizing plants. It binds at least 14 chlorophylls and carotenoids such as lutein and neoxanthin, as well as magnesium, and as such maintains the structural integrity and functionality of the complex.

This study was inspired by observing the widespread use of VA(aq) as a malarial medicine in Uganda, with the same preparation methodology being used for this work. The hypothesis that there may be an additional efficacious agent, other than sesquiterpene lactones, present in the preparation was owing to the appearance of artemisinin resistant strains of *Plasmodium falciparum*^22^; if the observed effects were due to vernodalin, then resistance would likely have occurred over the length of time the extract has been used by humans and primates. This lack of resistance also explains why we assessed for an immuno-modulatory factor; indirect targeting through host immunity does not apply the same selection pressure as a single direct targeting agent.

Immuno-modulatory studies resulted in the unexpected finding that VA(aq) bound to active TGFβ1. This was shown to be a shared feature amongst most plant species tested, with VA(aq) showing the greatest binding per mass. VA(aq) was further shown to neutralize the function of active TGFβ1 in a cellular system. Precipitation using TGFβ1 as a bait protein revealed a binding agent to be chlorophyll a-b binding protein (CabBP) AB96. Purified CabBP AB96 was then shown to bind to, and neutralize the function of, active TGFβ1.

## Results

**Figure 1:**
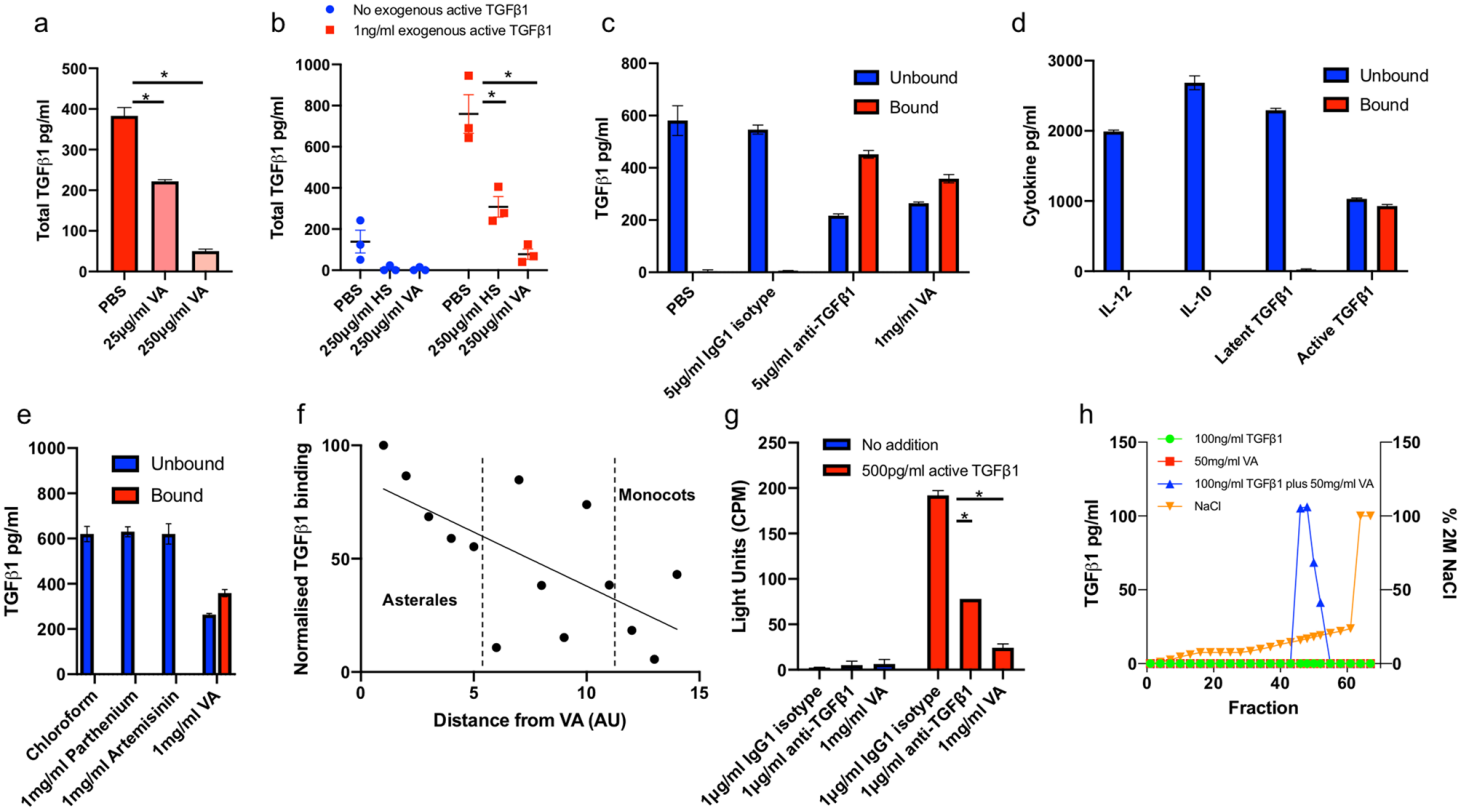
VA aqueous extract [VA(aq)] contains an active TGFβ1 neutralizing agent. The effects of VA extract on endogenous (**a**) and exogenous (**b**) TGFβ1 levels in the supernatant of monocyte derived dendritic cells. n = 1 for a; n = 3 for b. (**c**) Unbound (remaining in supernatant after incubation) and bound (liberated after washing, acidification and neutralization) active TGFβ1 concentrations (starting conc.: 2 ng/ml) after incubation with VA(aq) for 2 h at room temperature (RT). Representative of 3 independent assays. Technical triplicates. Mean +/− SEM shown. (**d**) The unbound and bound IL-10, IL12, latent TGFβ1 and active TGFβ1 concentrations (starting conc.: 4 ng/ml) after incubation with VA(aq) for 2 h at RT. Technical triplicates. Mean +/− SEM shown. (**e**) The unbound and bound active TGFβ1 concentrations (starting conc.: 2 ng/ml) after incubation with sesquiterpene lactones and VA(aq) for 2 h at RT. Technical triplicates. Mean +/− SEM shown. (**f**) The levels of active TGFβ1 bound to aqueous extracts (1 mg/ml) from different species of plants [normalized to VA(aq)] when incubated with 2 ng/ml active TGFβ1. Species in order of x axis: (1) *Vernonia amygdalina* Del. (Compositae). (2) *Vernonia arkansana* DC. (Compositae). (3) *Helianthus annuus* L. (Compositae). (4) *Aster dumosus* Hoffm. (Compositae). (5) *Campanula lactiflora* M.Bieb. (Campanulaceae). (6) *Petroselinum crispum* (Mill.) Fuss (Apiaceae). (7) *Ocimum basilicum* L. (Lamiaceae). (8) *Solanum lycopersicum* L. (Solanaceae). (9) *Spinacia oleracea* L. (Amaranthaceae). (10) *Rosa sericea Wall.* Ex Lindl. (Rosaceae). (11) *Vitis vinifera* L. (Vitaceae). (12) *Dieffenbachia amoena* Bull. (unresolved, Araceae) (13) *Dracaena marginata* Hort. (Asparagaceae). (14) *Lilium candidum* L. (Liliaceae). Technical triplicates. Mean +/− SEM shown. (**g**) The effect of VA extract on luciferase activity in a functional active TGFβ1 reporter cell line. Biological triplicates. Mean +/− SEM shown. Representative of 2 independent assays. (**h**) The levels of TGFβ1 in different fractions eluted from an anion exchange column after loading with indicated factors, using indicated % of 2 M NaCl. Unpaired student’s t-test: *p < 0.05.

The immunological effects of endotoxin-free aqueous extract of VA [VA(aq)], on human monocyte derived dendritic cells (mo-DCs) was studied. Mo-DCs were incubated with VA(aq) for 24 h after which they were assessed for changes in cell surface proteins and cytokine secretion. Although minor mo-DC activation was observed when assessing cell surface markers such as CD83 and CD86 by flow cytometry (data not shown), the most profound change was found to be in the supernatant where treated cells were shown to have significantly lower concentrations of total (active and latent) TGFβ1 (Fig. 1a). Our initial hypothesis was that this indicated a specific inhibition of TGFβ1 production, however when exogenous active TGFβ1 was added to the system, we saw the same reduction. This occurred in a similar manner to a known active TGFβ1 binder, heparin sulphate (HS)^23^ (Fig. 1b), suggesting that an agent in the extract may be binding and ‘removing’ active TGFβ1 from the culture supernatant.

To assess if VA extract was binding to active TGFβ1 we developed a simple in vitro method whereby we plated out VA(aq) overnight, before washing and blocking. We then incubated the blocked VA(aq) with active TGFβ1 for 2 h. After incubation the supernatant was removed from the plate for testing (the ‘unbound fraction’). The plate was then washed, acidified and neutralized, to elute the ‘bound fraction’. Active TGFβ1 was measured in the unbound and bound fractions, with bound TGFβ1 seen in the VA(aq) and an anti-TGFβ1 antibody wells (Fig. 1c). We were next concerned that this binding may be non-specific and so tested 3 other cytokines, IL-10, IL-12p70 and, importantly, latent TGFβ1, and found that this phenomenon could only be observed with active TGFβ1 (Fig. 1d).

We then considered if sesquiterpene lactones, common efficacious agents in medicinal plants and present in VA, may be responsible for the observed binding. We assayed both artemisinin and parthenium finding that neither were able to bind active TGFβ1 under these conditions (Fig. 1e). Subsequently, we wanted to explore if VA(aq) was unique in having this TGFβ1 binding ability. We prepared extracts from 13 additional species, representing different branches of the plant kingdom and tested their ability to bind active TGFβ1. All species were able to bind, however there was a trend suggesting that members of the Asterales order, which contains many medicinal plants, contained more of the active TGFβ1 binding compound, whilst the Monocot clade contained less. (Fig. 1f). We next wished to assess if this binding rendered active TGFβ1 non-functional. Using a well-established reporter assay (where luciferase runs off the promotor of the TGFβ1 target SERPINE1 (PAI-1; plasminogen activator inhibitor-1)^24^), we were able to show that VA(aq) was able to inhibit active TGFβ1 function (Fig. 1g).

We next used anion exchange chromatography to show a) that binding was occurring using a different assay and b) to suggest the isoelectric point of the agent for validation purposes. As can be seen in Fig. 1h, active TGFβ1 was again seen to bind to VA(aq) (with elution of active TGFβ1 only occurring with pre-incubation) and, in combination with the agent, active TGFβ1 eluted in fractions 46–52, which given the experimental conditions, gave the agent an isoelectric point of 4.5–5.5.

**Figure 2:**
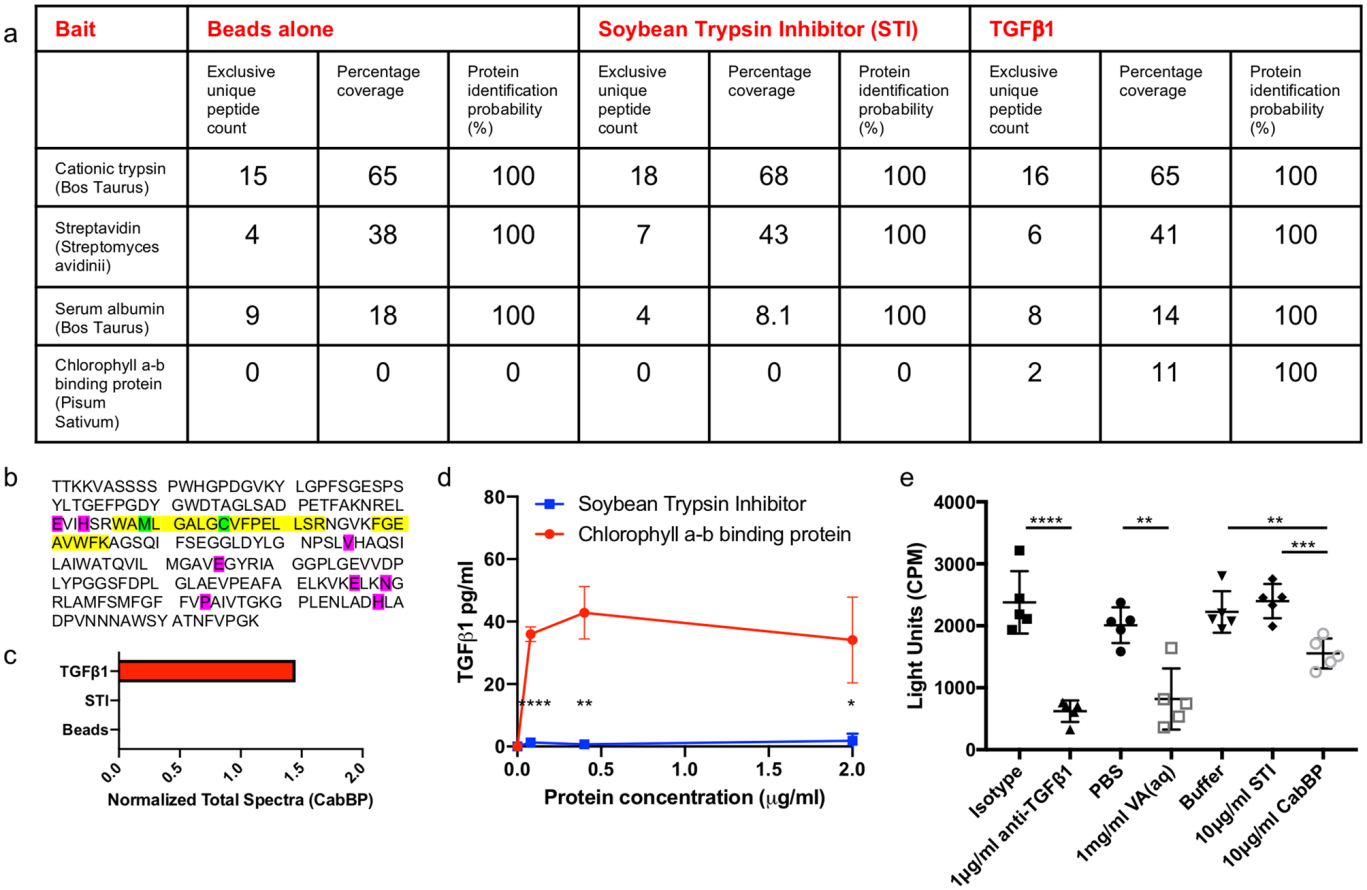
Chlorophyll a-b binding protein (CabBP) AB96 binds to and neutralizes active TGFβ1. (**a**) Analyzed mass spectrometry data showing pulled out proteins when using indicated bait proteins when a threshold of min number of peptides = 2, protein threshold = 20% and peptide threshold = 95%. (**b**) The chlorophyll a-b binding protein AB96 peptides from *Pisum Sativum* (Fabaceae), observed by mass spectrometry are highlighted in yellow. Green represents amino acid modifications: M, oxidation and C, carbamidomethyl. Pink represents Magnesium binding sites. (**c**) Quantitation of chlorophyll a-b binding protein AB96 pulled from VA(aq) using indicated bait protein. (**d**) Concentration of active TGFβ1 eluted (bound fraction) from different concentrations of chlorophyll a-b binding protein AB96 (CabBP) or Soybean trypsin inhibitor (STI) control after incubation for 2 h at RT. Mean +/− SEM shown. Example of 2 independent experiments (**e**) The effect of VA(aq), anti-TGFβ1 antibody, CabBP, STI and relevant controls, on luciferase activity in an active TGFβ1 reporter cell line. Biological quintuplets. Mean +/− SEM shown. Unpaired student t-test. *p < 0.05; **p < 0.01; ***p < 0.001; ****p < 0.0001.

To isolate the active agent we performed a modified immunoprecipitation (IP), incubating VA(aq) with biotinylated active TGFβ1, or control protein (soybean trypsin inhibitor; STI) and streptavidin beads. The eluant was run on a gel and whole lanes were sent for mass spectrometric analysis. Two peptides were obtained, EVIHSRWAMLGALGCVFPELLSR and FGEAVWFK which are both present in the same protein; chlorophyll a-b binding protein (CabBP) AB96 (P04159). The initial hits were identified as being part of the sequence from *P. sativum* L. (Leguminosae)*,* (Fig. 2a–c) but with the sequence for VA unavailable at this current time and the protein well-conserved, it is assumed that this sequence is also present in VA. These peptides were seen to have bound active TGFβ1 and not the controls (Fig. 2a,c). Using *E.coli* derived recombinant full-length folded CabBP AB96 from *P. sativum* L. (Leguminosae)*,* we repeated the binding assays finding that CabBP AB96 was able to bind active TGFβ1 (Fig. 2d). Finally we repeated the functional assay using the luciferase reporter system, finding that CabBP AB96 was also able to inhibit the function of TGFβ1 in a significant manner when compared to either STI or CabBP buffer (Fig. 2e).

## Discussion

This study reveals the first plant-derived cytokine binding protein. The central role that active TGFβ1 plays in many of the pathologies VA and other medicinal plants are ingested for, suggests that this protein, or a peptide derived from this protein, may help to explain some of the observed effects.

One of our chief concerns with this work is the fact that monocots do not appear to be able to bind active TGFβ1 well, despite expressing CabBP. This is likely to be due the increased amount of vascular tissue in monocots compared to eudicots, meaning that, proportionally, less chlorophyll is present. Given our extract concentrations were based on total mass we believe it would be reasonable to conclude our results were due to these much lower amounts of CabBP in the starting material.

CabBP AB96 is membrane bound and therefore difficult to isolate without detergents and sonication. It is likely that the preparation methodology, which was robust and includes the use of detergents to remove endotoxin, allowed for its presence in the extracts. Indeed, with one of the peptides overlapping a transmembrane region (WAMLGALGCVFPELLSR; underlined region shows overlap) we believe that this must have occurred.

Although we have shown that the whole recombinant *E. coli* produced protein can bind TGFβ1, we do not yet know if the whole protein is required for this binding. The peptides picked up in the mass spectrometry are next to each other, and may be suggestive of a functional region, however further studies are required to confirm whether or not this is the case.

The question remains as to why CabBP AB96 has the ability to bind active TGFβ1. We feel the answer may lie, in part, in relative charges; active TGFβ1 is more positively charged (isoelectric point; 7.73) and this charge is critical for its functionality in maintaining latency, whilst CabBP AB96 is more negatively charged (isoelectric point; 4.88) enabling it to bind magnesium ions (along with chlorophyll, lutein and neoxanthin). This could place CabBP AB96 as a heparin-mimetic, acting as a scaffold to cause the oligomerization of TGFβ1^23^. However, although charge may be important, it is unlikely that this is the sole determinant. Binding studies using more sophisticated techniques (e.g. isothermal titration calorimetry or surface plasmon resonance) are required to fully answer this question.

Whether CabBP AB96 can have a real impact on pathology, depends on the protein, or peptide’s, functional survival after acidification, neutralization and digestion in the gastrointestinal tract, and entry into the circulation. Our mass spectrometry data show that the protein can be degraded by trypsin, however there is currently limited evidence of plant peptides entering and remaining in the circulation^25^. For intestinal helminth infections, there are fewer delivery concerns as CabBP AB96 peptides could act directly, or close to, the site of pathology without gaining entry into the circulation.

Regarding the reported efficacy of VA extract itself, the answer may lie in a combination of agents that act either directly on the pathogen (e.g. sesquiterpene lactones) or indirectly via immune polarization (e.g. β-glucans) or removal of immune evasion (CabBP AB96). We now know enough about pathogenic mutation under selection pressure to understand that a single agent is unlikely to be efficacious for the length of time VA appears to have been used.

In conclusion, CabBP AB96 has been shown to bind to and inactivate active TGFβ1. The next step will be to identify whether this is a property of the whole protein, or merely a peptide fragment. In the case that this activity can be localized, then it may be possible to manufacture a mimetic or a peptide which carries the same activity, and this would have potential for therapeutic intervention.

## Methods

### Preparation of leaf extracts

Defined species were sourced from UK horticultural suppliers. These species are common, not endangered and therefore are not subject to the IUCN Policy Statement on Research involving species at risk of extinction, or the Convention on the trade in endangered species of wild fauna and flora. The species were validated by the suppliers. Fresh leaves were pulped with 50 ml distilled water using a pestle and mortar. The extracts were filtered (45 nm), before being boiled for 30 min and re-filtered (45 nm). Samples were then subjected to three endotoxin clean-ups [until the LAL test proved negative], using Triton-X114 (Sigma) as described^26^. The samples were then quantified either using a spectrophotometer or BCA assay (Pierce). Endotoxin tests (LAL; Lonza) were carried out in the presence of a β-glucan block (Lonza) [β-glucans present in plant cell walls cross react in the LAL assay] and samples were only used if levels were < 0.1EU/mg.

### Generation of monocyte-derived dendritic cells

Blood packs were acquired from the National Health Service Blood and Transplant Service (NHSBTS) an HTA licenced provider. Monocytes were isolated using CD14 microbeads (Miltenyi Biotec) and the MACS system (Mitenyi Biotec). After treatment with 1500U/ml IL4 (Bio-Techne) and 400U/ml GMCSF (Bio-Techne), cells were cultured in AIM-V media (GIBCO) for 6 days. Cells were confirmed to be immature DCs using flow cytometry as described^27^. Extract was diluted in PBS to reach stock concentration for use on cells. Heparin sulphate (Sigma) was dissolved in PBS before use. Cell viability was assayed using Annexin-V/PI (BD biosciences) staining by flow cytometry.

### Protein binding assay

Leaf extract (1 mg/ml in PBS), TGFβ1 antibody (5 µg/ml in PBS; clone 1D11 Bio-Techne), parthenium (1 mg/ml in chloroform; Sigma), artemisinin (1 mg/ml in chloroform; Sigma), full length *Pisum sativum* L. (Leguminosae) chlorophyll a-b binding protein AB96 (at indicated concentrations; mybiosource TTKKVASSSSPWHGPDGVK YLGPFSGESPSYLTGEFPGDY GWDTAGLSADPETFAK NRELEVIHSRWAMLGAL GCVFPELLSRNGVKFG EAVWFKAGSQIFSEGGL DYLGNPSLVHAQSIL AIWATQVILMGAVEGYRI AGGPLGEVVDPLYPG GS FDPLGLAEVPEAFAE LKVKELKNGRLAMF SMF GFFVPAIVTGKGPLEN LADHLADPVNNN AWSYATNFVPGK), soybean trypsin inhibitor (at indicated concentrations; Sigma) or vehicle (PBS or chloroform) were plated on a high binding 96 well flat-bottomed plate (Greiner) and left at RT overnight. The next day the plate was washed × 3 in PBS + 0.05% Tween-20 (Sigma) before being blocked with 5% Tween-20 in PBS. Active TGFβ1 (Bio-Techne), IL-10 (Bio-Techne), IL-12p70 (Bio-Techne) or latent TGFβ1 (Bio-Techne) were added at indicated concentrations before being incubated for 2 h at RT. The supernatant was removed and placed immediately in a relevant ELISA for assaying; this formed the ‘unbound fraction’. The plate was washed × 3 in PBS + 0.05% Tween-20 before being treated with 50 µl 1 M HCl for 30 min at RT to elute any bound protein. This ‘bound fraction’ was neutralised using 50 µl 1.2 M NaOH/0.5 M HEPES and immediately assayed in a relevant ELISA.

### ELISAs

TGFβ1, IL-12 and IL-10 ELISAs (all Bio-Techne) were performed as per the manufacturer’s instructions.

### TGFβ1 reporter assay

MINK cells (generously provided by Professor Rifkin) were cultured and assays performed as described^28^. Antibodies, VA extract, CabBP, STI and CabBP buffer were diluted in PBS prior to use. Cell viability was assayed using trypan blue after the assay period.

### Anion exchange chromatography

Samples were prepared as follows: 10 ml 100 ng/ml active TGFβ1 (Bio-Techne) in PBS. 10 ml 50 mg/ml VA extract in PBS. 5 ml 100 ng/ml active TGFβ1 in PBS plus 5 ml 50 mg/ml VA extract in PBS, mixed for 4 h on a rotator at RT. Samples were loaded using a 50 ml Superloop (Cytiva) and run on an ACTApurifier 10 system (Cytiva) using the equipment, reagents and programme described^29^. Fractions were assayed for TGFβ1 using an ELISA as described.

### Modified immunoprecipitation

500 µl 1 µg/ml biotinylated active TGFβ1 in PBS (Bio-Techne), 500 µl 1 µg/ml biotinylated Soybean Trypsin Inhibitor (Bio-Techne) and PBS were each mixed with 500 µl of 50 mg/ml VA extract in PBS on a rotator for 4 h at RT. 50 µl magnetic streptavidin beads (Pierce) were washed in TBS + 0.1% Tween-20, added to each mixture, and mixing continued for a further 2 h. Beads were pulled out using a magnetic stand and samples were washed × 3 in TBS + 0.1% Tween-20. Samples were eluted from beads using SDS-PAGE reducing sample buffer (ThermoFisher) and run on SDS PAGE (ThermoFisher). Gels were stained and destained using a silver staining for mass spectrometry kit (Pierce). Whole lanes were cut out and analysed using mass spectrometry.

### Mass spectrometry

Gel sections were digested with bovine trypsin (Sigma) following reduction and alkylation of cysteine bonds with dithiothreitol and iodoacetamide (Sigma). Peptide extracts were subjected to chromatographic separation by C18 reversed-phase nano-trapping (Acclaim PepMap100 C18 Trap, 5 mm × 300 µm) and nano-analytical columns (EASY-Spray PepMap C18, 2 μm 100 Å, 75 µm × 15 cm) on an EASY NanoLC system (ThermoFisher) using a three-step linear gradient at a flowrate of 250 nl/min over 60 min. The eluate was ionized by electrospray ionization using an Orbitrap Velos Pro (ThermoFisher) operating under Xcalibur v2.2. Precursor ions were selected according to their intensity using the collision-induced fragmentation employing a Top20 CID method. Raw spectral data was processed using Proteome Discoverer (v1.4) against the Uniprot ‘All Taxonomy’ database under the Mascot 2.2 algorithm (Matrix Science). Results were analysed using Scaffold Software (version 4.11.0; Proteome Software).

This study was carried out in compliance with relevant institutional, national, and international guidelines, and legislation.

## Data Availability

The datasets generated during and/or analyzed during the current study are available from the corresponding author on reasonable request.
